# *In vitro* comparative analysis of steamed wood and other lignocellulosic substrates on ruminal fermentation and microbiota

**DOI:** 10.3389/fvets.2026.1750760

**Published:** 2026-01-30

**Authors:** Kazuaki Ito, Makoto Adachi, Andrew J. Scheftgen, Garret Suen, Ryo Hiyama, Kazuto Seki, Shintaro Nakai, Rintaro Yano, Takehiro Nishida, Masaaki Hanada, Naoki Fukuma

**Affiliations:** 1 Graduate School of Animal and Veterinary Sciences and Agriculture, Obihiro University of Agriculture and Veterinary Medicine, Obihiro, Hokkaido, Japan; 2 Department of Bacteriology, University of Wisconsin-Madison, Madison, WI, United States; 3 Hokkaido Research Organization, Forest Products Research Institute, Asahikawa, Hokkaido, Japan; 4 ACE-CLEAN Co., Ltd., Kitami, Hokkaido, Japan; 5 Department of Life and Food Sciences, Obihiro University of Agriculture and Veterinary Medicine, Obihiro, Hokkaido, Japan; 6 Research Center for Global Agromedicine, Obihiro University of Agriculture and Veterinary Medicine, Obihiro, Hokkaido, Japan

**Keywords:** cattle, microbiome, roughage, steaming, wood, xylo-oligosaccharides

## Abstract

Lignocellulosic biomass such as wood is increasingly recognized as a promising low-opportunity-cost feed (LCF) that does not compete with human-edible food. In this study, we evaluated rumen fermentation characteristics and microbial community responses using an *in vitro* batch culture system with a diverse set of substrates, including steamed and untreated woods, xylo-oligosaccharides, spent mushroom substrates, and conventional feeds. Hierarchical clustering based on bacterial community composition revealed five distinct microbial clusters. Certain steamed woods and xylo-oligosaccharides formed separate clusters from untreated woods and conventional feeds, and were associated with the dominance of specific genera such as *Succinivibrio* and *Selenomonas*. These microbial shifts may reflect differences in substrate characteristics, potentially related to hemicellulose- and *oligosaccharide*-derived components. The results suggest that both steamed wood and xylo-oligosaccharides enhance fermentability and are associated with distinct microbial community structures under *in vitro* conditions. These substrates show promise as sustainable feed ingredients, and further *in vivo* studies are needed to evaluate their efficacy and long-term impacts on animal health and productivity.

## 1 Introduction

With increasing global population growth, securing sustainable food production without expanding arable land is critically important. Currently, about one-third of arable land and global cereal output is used for livestock feed, leading to significant food-feed competition and conversion losses (1). To address these issues, feedstuffs that do not compete with human-edible food—often referred to as low-opportunity-cost feeds (LCFs), such as food by-products and grassland biomass have been proposed to improve food system efficiency and reduce environmental burdens (2, 3). Among LCFs, lignocellulosic biomass such as wood is particularly promising due to its abundance and independence from arable land (1, 4). For example, in temperate and cold zone regions in the northern hemisphere, birch species (*Betula* spp.) are widely distributed, with three species—Japanese white birch (*B*. *platyphylla* var. japonica), Erman's birch (*B. ermanii*), and Japanese red birch (*B*. *maximowicziana*)—being commercially important in Hokkaido, Japan due to their large stocks and material production volumes (5). However, the by-products of birch wood production are largely underutilized, primarily being used as sawdust for mushroom cultivation or for disposable wooden products in local industries (5). Utilizing a portion of these underused resources under appropriate conditions could contribute to circular bioeconomy efforts in livestock systems.

However, high lignin content in wood is a primary limitation to ruminal digestibility (6) because lignin forms a complex network with carbohydrates that hinders microbial access, and thus cannot be degraded in the anaerobic rumen environment of typical ruminants (4, 7). Accordingly, fermentation of woody biomass in the rumen does not involve lignin itself, but rather depends on the accessibility of non-lignin carbohydrate fractions. To enhance its nutritional value, appropriate pre-treatment methods are essential (4) and various approaches have been explored to improve lignocellulosic digestibility. Among them, biological pretreatment using white rot fungi, such as *Lentinula edodes*, has received attention for its ability to selectively degrade lignin and enhance carbohydrate availability to rumen microbes (4, 6). However, fungal pretreatment generally requires a relatively long incubation period, which can limit its practical applicability in large-scale operations (6).

Alternatively, steam treatment rapidly alters the lignocellulosic matrix through high temperature and pressure, thereby improving microbial access to non-lignified polysaccharide fractions. For example, Kajikawa et al. (8) demonstrated that replacing alfalfa hay cubes with steamed white-birch (30% and 60% of the diet on a dry matter basis) increased daily weight gain of Holstein steers from 1.23 to 1.34 kg/day. Additionally, Hiyama et al. (9) reported that replacing fermented bagasse with steamed white-birch increased carcass weight in Japanese Black steers by approximately 5% compared to the controls. These findings suggest that steamed lignified biomass may serve as an alternative roughage source, provided that large-scale steaming is economically and ecologically viable, although studies on its use remain limited. During steam treatment, components of wood fibers are degraded into oligosaccharides, which are often used as prebiotics and are known to influence specific bacteria and their fermentation activity (10). For example, Michalak et al. (11) reported that feeding oligosaccharides derived from Norway spruce (*Picea abies*) to piglets increased the relative abundance of butyrate-producing bacteria such as *Roseburia* spp. This finding suggests that oligosaccharides generated through steam treatment may modulate the rumen microbiome.

Regulation of the ruminal microbiota and fermentation has gained increasing attention in recent years as a key strategy not only for methane mitigation but also for supporting rumen health. Given this context, steamed wood may have dual potential—not only as a sustainable roughage source but also as a functional modulator of microbial community structure in the rumen. These findings collectively suggest that woody biomass may possess biological functions beyond basic nutritional value. Furthermore, by elucidating the potential of these woody substrates as viable feed ingredients, this study could contribute to the development of integrated feed production strategies that link forestry, mechanical engineering, and livestock production. To further explore these functional properties, we conducted a comparative *in vitro* culture test to evaluate differences in fermentability and bacterial community structure among underused lignocellulosic biomasses, including steamed woods, non-steamed form, spent mushroom substrates, and steam-derived oligosaccharides.

## 2 Materials and methods

### 2.1 Substrate

In this study, 90 mg dry matter of 25 substrates were used for *in vitro* incubation: six common feeds (timothy hay, timothy hay, and concentrate (1:1, DM basis), concentrate, rice straw, oats hay, and fermented bagasse), four types of wood meal [white-birch wood (*Betula platyphylla*), willow wood (mixture of *Salix sachalinensis* and *S. schwerinii*), larch wood (*Larix kaempferi*), and fir wood (*Abies sachalinensis*)], four types of steamed wood (steamed white-birch, willow, larch, and fir), three spent mushroom substrates (*Lentinula edodes, Grifola frondosa*, and *Flammulina velutipes*), and eight types of oligosaccharides (white-birch oligosaccharide, willow oligosaccharide, xylo-oligosaccharide, xylo-oligosaccharide neutral fraction, xylo-oligosaccharide acidic fraction, bamboo-culm oligosaccharide, bamboo-culm oligosaccharide powder, and xylan). Detailed information on the origin, preparation methods, and characteristics of the substrates used in this experiment are provided in Table 1. The concentrate used in this study was a commercially available corn-based concentrate (20.5% crude protein, 5.0% crude ash, 2.6% ether extract, 28.5% neutral detergent fiber, 6.7% acid detergent fiber, and 43.4% non-fiber carbohydrate; all on a dry matter basis).

**Table 1:** Details of substrates used in *in vitro* fermentation experiments, including origin, processing method, and composition.

**Name/source material**	**Processing method**	**Supplier/origin**	**Notes (e.g., solid content, particle size)**
Timothy hay	—	—	—
Timothy hay and concentrate	—	Snow Brand Seed Co., Hokkaido, Japan	—
Concentrate	—	Snow Brand Seed Co., Hokkaido, Japan	—
Rice straw	—	—	—
Oats hay	—	—	—
Fermented bagasse	—	BIO Science Co., Tokushima, Japan	—
White birch (*B*. *platyphylla*)	Milled, 1 mm screen	—	—
Willow (*S*. *sachalinensis*, S. *schwerinii*)	Milled, 1 mm screen	—	—
Larch (*Larix kaempferi*)	Milled, 1 mm screen	—	—
Fir (*Abies sachalinensis*)	Milled, 1 mm screen	—	—
White birch (*B*. *platyphylla*)	Steam: 180 °C, 10 min; then milled (1 mm pass)	—	—
Willow (*S*. *sachalinensis*, S. *schwerinii*)	Steam: 180 °C, 10 min; then milled (1 mm pass)	—	—
Larch (*Larix kaempferi*)	Steam: 200 °C, 20 min; then milled (1 mm pass)	—	—
Fir (*Abies sachalinensis*)	Steam: 200 °C, 20 min; then milled (1 mm pass)	—	—
*L*. *edodes*	Milled (1 mm); wood substrate: birch + larch	—	Substrate: birch and larch wood
*G*. *frondosa*	Milled (1 mm); wood substrate: willow	—	Substrate: willow wood
*F*. *velutipes*	Milled (1 mm); substrate: corn cob	—	Substrate: corn cobs
Birch *oligosaccharide*	Steam: 180 °C, 30 min → extract → concentrate	—	35 wt% solid content
Willow *oligosaccharide*	Steam: 200 °C, 10 min → extract → concentrate	—	65 wt% solid content
*Xylo-oligosaccharide*	—	Suntory Beverage & Food Ltd., Osaka, Japan	70 wt% solid content
*Xylo-oligosaccharide* neutral fraction	—	Oji Paper Co. Ltd., Tokyo, Japan	—
*Xylo-oligosaccharide* acidic fraction	—	Oji Paper Co. Ltd., Tokyo, Japan	Acidic sugar fraction containing uronic acids, particularly glucuronic acid side chains.
Bamboo-culm *oligosaccharide* (*S*. *senanensis*)	Steam: 200 °C, 10 min → extract → concentrate	—	35 wt% solid content
Bamboo-culm powder (*S*. *senanensis*)	Steam: 200 °C, 10 min → extract → spray-dry	Ohkawara Kakohki Co., Yokohama, Japan	Spray drying machine (L-8 AS, Ohkawara Kakohki, Co., LTD, Yokohama, Japan)
Oat spelt xylan	—	Tokyo Chemical Industry Co., Tokyo, Japan	—

### 2.2 Rumen fluid collection

Rumen fluid was collected at 1:00 p.m. from two healthy, fistulated, 6-year-old Holstein dry cows (average body weight: 880 kg) that were offered timothy hay *ad libitum* at the Field Center of Animal Science and Agriculture, Obihiro University of Agriculture and Veterinary Medicine (Obihiro, Hokkaido, Japan). Rumen fluid (300 ml per cow) was collected from four locations within the rumen, and samples from both cows were mixed and filtered through four layers of gauze. The mixed rumen fluid was immediately transferred into closed bottles, and maintained at 39 °C until *in vitro* batch cultures could be performed. For subsequent experiments, the remaining rumen contents were stored at −20 °C. Animal management and sampling procedures were approved by the Animal Care and Use Committee of the Obihiro University of Agriculture and Veterinary Medicine (Approval ID: 21-177).

### 2.3 *In vitro* culture test

*In vitro* cultures were incubated at 39 °C for 18 h. Artificial saliva (12) and strained rumen fluid were mixed in a 1:2 ratio, and the pH of the artificial saliva was adjusted to 6.8 by continuous CO_2_ bubbling prior to incubation. A total of 9 ml of the mixture was aliquoted into three replicate Hungate tubes with corresponding substrates. Anaerobic conditions were maintained by flushing the artificial saliva with CO_2_ gas prior to use. During filling, Hungate tubes were continuously flushed with CO_2_ to minimize oxygen exposure and were immediately sealed. No chemical reducing agents were used. Tubes were mixed prior to incubation but were not agitated during the incubation. After 18 h of incubation, total gas production was measured with a 50 ml syringe. In addition, pH of the cultures was measured with a pH meter (LAQUA F-72, HORIBA, Ltd., Kyoto, Japan) and 1 ml of the cultures were collected and stored in a 1.5 ml tube for subsequent analyses. Cultures were centrifuged, after which the supernatant was diluted two times with distilled water, and the concentration of formate, lactate and other SCFAs were monitored using HPLC as described previously (13). The pellet obtained after centrifugation was used for DNA extraction for microbial analysis.

### 2.4 Microbial analysis

Mechanical disruption of the cells was performed using the repeated bead beating plus column (RBB + C) method (14). DNA extraction was performed using the Maxwell^®^ RSC Blood DNA Kit (Promega Corporation, Madison, WI, USA). The concentration and purity of extracted DNA was measured using a QuantusTM Fluorometer and the QuantiFluor^®^ ONE dsDNA System (Promega Corporation, Madison, WI, USA). DNA was diluted to a final concentration of 5 ng/μl using Tris-EDTA buffer. Bacterial sequencing was performed via two-step amplification of the V3–V4 regions of the 16S rRNA gene using primers consisting of Illumina overhang adapters and universal bacterial primers (F-TCGTCGGCAGCGTCAGATGTGTATAAGAGACAGCCTACGGGNGGCWGCAG; R-GTCTCGTGGGCTCGGAGATGTGTATAAGAGACAGGACTACHVGGGTATCTAATCC). In the second PCR, Illumina sequencing adapters and dual index barcodes were added to the amplicons using a Nextera^®^ XT Index Kit (Illumina Inc., San Diego, California, USA). The concentration of the second PCR product was quantified using a QuantusTM Fluorometer and the QuantiFluor^®^ ONE dsDNA System and libraries for each amplicon type were created using an equimolar (4 nm) pool of all PCR products. The resulting bacterial and archaeal amplicons were sequenced on an Illumina MiSeq, using the MiSeq Reagent Kit v3 600 Cycles PE (Illumina, San Diego, CA, USA). Nucleotide sequence data is available on the DNA Data Bank of Japan's Sequence Read Archive (DRR699410-DRR699505). An amplicon sequence variant (ASV) table, taxonomic classifications, and a rooted phylogenetic tree were generated using QIIME2 version 2022.2 (15). ASVs were inferred using the DADA2 plugin (16) implemented in QIIME2. The ASV table, taxonomy file, and rooted tree were imported into R (version 4.2.3) using the qiime2R package for downstream analysis (17). Taxonomy was assigned using the SILVA 16S rRNA gene database version 138.1 (18), and the phylogenetic tree was constructed using the SEPP algorithm implemented in QIIME2.

### 2.5 Statistical analysis

Rumen fermentation and microbial profile data were statistically analyzed using GraphPad Prism 10.1.1 (GraphPad Software, San Diego, CA, USA). Statistical comparisons between groups were calculated and assessed using the Kruskal–Wallis rank-sum test (19), and further pairwise comparisons were performed using Dunn's multiple comparisons test (20). Alpha diversity was computed using QIIME2 (version 2022.2) with all samples rarefied to the minimum read count observed among them. Rarefaction was performed once at a fixed sequencing depth prior to diversity analyses. Principal coordinate analysis (PCoA) and hierarchical clustering based on weighted UniFrac distances were performed using R version 4.4.1 (21) with the packages phyloseq (22), microbiome (23), ggplot2 (24), stats, ggdendro (25), and RColorBrewer (26) to evaluate differences in microbial community composition between groups. To assess relationships between microbial community composition and fermentation parameters, envfit analysis was performed using the vegan package (27) in R. Fermentation parameters, including total SCFA concentration, individual volatile fatty acids, and total gas production, were fitted onto the weighted UniFrac-based PCoA ordination, and statistical significance was evaluated using 999 permutations. Additionally, differential abundance analysis was carried out using Linear Discriminant Analysis Effect Size (LEfSe) (28) via the microbiomeMarker package (29), with a *P*-value threshold of 0.05 and an LDA score cutoff of 3.0 to identify taxonomic biomarkers.

## 3 Results

**Figure 1:**
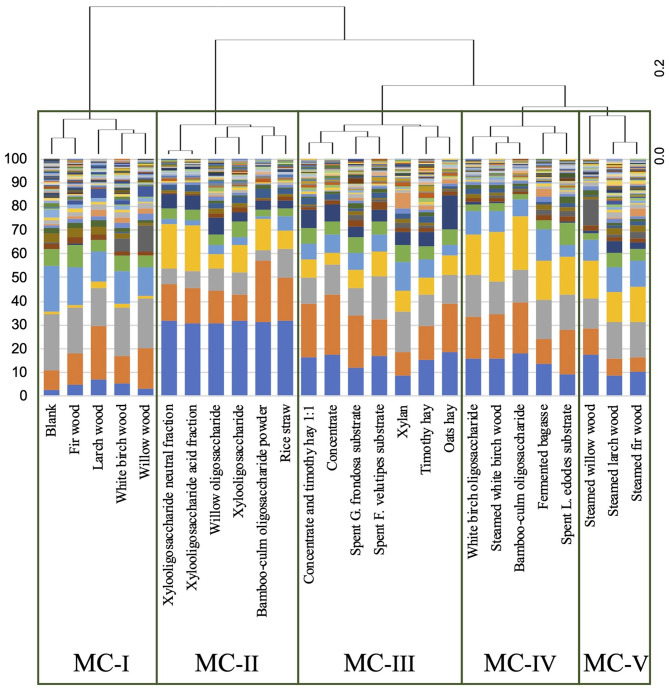
Hierarchical clustering of bacterial communities across different feed substrates. Hierarchical cluster analysis based on weighted UniFrac was used to identify distinct microbial community (MC) groups across fermentation samples.

**Figure 2:**
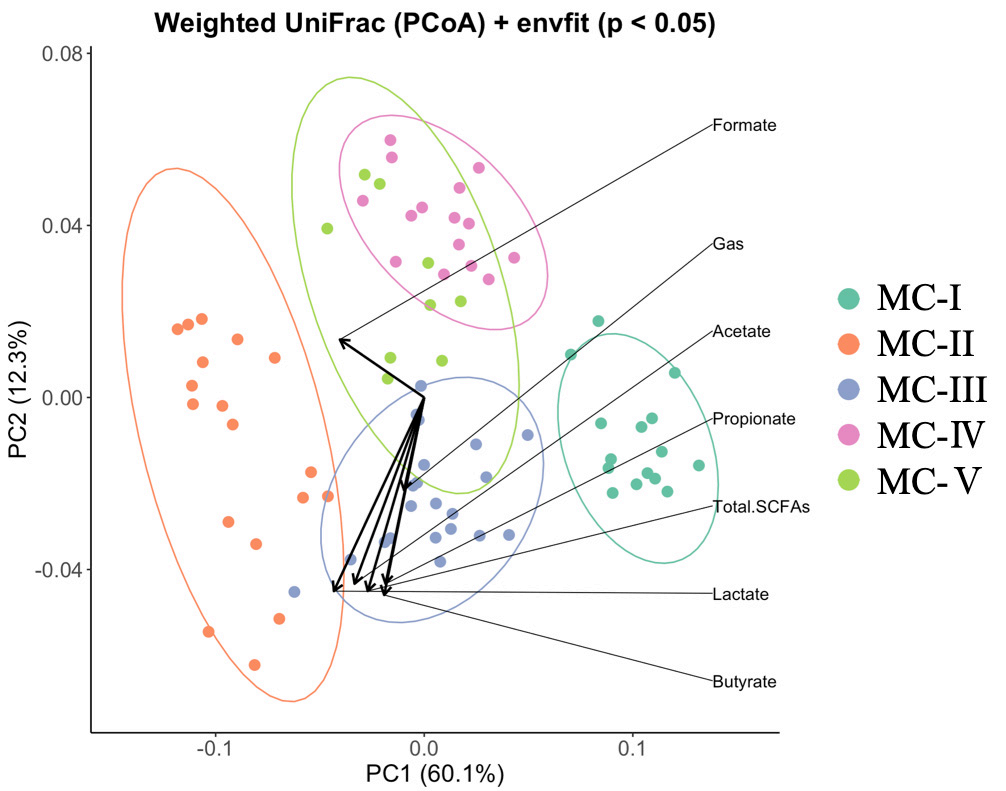
Principal coordinate analysis (PCoA) of ruminal microbial communities. PCoA was performed using weighted UniFrac distances to visualize beta diversity among samples. Each point represents an individual fermentation replicate, and colors indicate the assigned microbial community (MC) group. Vectors represent fermentation parameters fitted onto the ordination using envfit analysis, indicating their relative associations with the PCoA axes.

**Figure 3:**
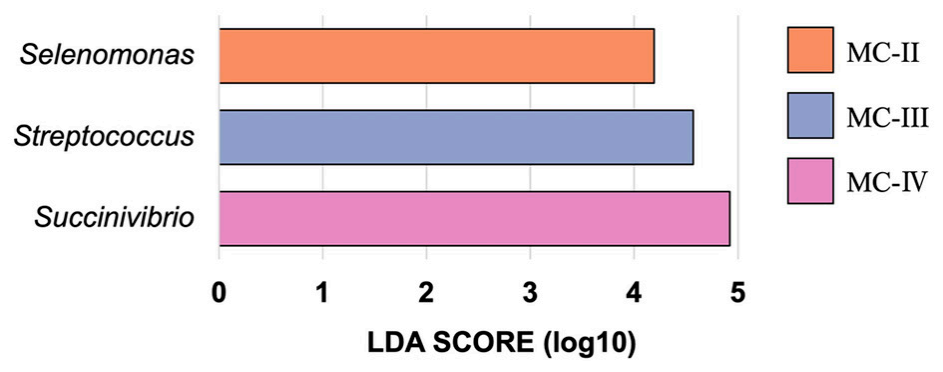
Genus-level taxonomic biomarkers identified by LEfSe analysis. Differential abundance analysis was performed using Linear Discriminant Analysis Effect Size (LEfSe) to identify bacterial genera that were significantly different among microbial community (MC) groups. A significance threshold of *P* < 0.05 and an LDA score cutoff of 3.0 were applied.

Bacterial sequencing yielded an average of 15,046 ± 6,974 high-quality, filtered reads containing an average of 230 ± 102 ASVs. We identified 46 genera that comprised >0.1% of the microbial communities across all substrates (Supplementary Table S1). Samples were grouped via hierarchical clustering using weighted UniFrac distances (Figure 1, 2) and were found to cluster into five different microbial communities (MCs). MC-I comprised those MCs grown on non-steamed woods (white-birch, willow, larch, and fir) and the blank. MC-II contained rice straw, three xylo-oligosaccharides (xylo-oligosaccharide, xylo-oligosaccharide neutral fraction and xylo-oligosaccharide acidic fraction) and two oligosaccharides (willow oligosaccharide and bamboo-leaf oligosaccharide powder). MC-III comprised four common feeds (timothy hay, timothy hay and concentrate 1:1, concentrate and oats hay), two spent mushroom (G. frondosa and F. velutipes) substrates and xylan. MC-IV comprised steamed white-birch, fermented bagasse, spent *L. edodes* mushroom substrate and two oligosaccharides (white-birch oligosaccharide and bamboo-culm oligosaccharide). MC-V comprised three steamed woods (willow, larch, and fir). To quantitatively evaluate the relationship between fermentation parameters and microbial community structure, envfit analysis was performed based on the weighted UniFrac ordination. Total SCFA concentration, gas production, and several individual VFAs were significantly correlated with the ordination (*p* < 0.05). The fitted vectors for total SCFAs, gas production, and most VFAs were more strongly aligned with PC2, whereas formate showed a stronger association with PC1, which explained 60.1% of the total variance (Figure 2 and Supplementary Table S2). The genera abundance data of each group is shown in Supplementary Table S3. Three genera, *Selenomonas, Streptococcus*, and *Succinivibrio*, were found as biomarkers associated with specific MC groups via LEfSe analysis (Figure 3). *Selenomonas* was enriched in MC-II, *Streptococcus* in MC-III, and *Succinivibrio* in MC-IV. No specific genus-level biomarkers were detected in MC-I and MC-V.

The results of α-diversity analysis are presented in Table 2. MC-I exhibited significantly higher Chao1 than MC-II (*p* < 0.0001), and MC-IV (*p* = 0.0172) and MC-V (*p* = 0.0002). No other group comparisons were significant. For Pielou evenness, MC-I showed significantly higher evenness than MC-II (*p* < 0.0001), MC-III (*p* = 0.0077), and MC-IV (*p* < 0.0001). Additionally, MC-II exhibited significantly lower values than MC-III (*p* < 0.0001) and MC-V (*p* = 0.0002). In Faith's phylogenetic diversity (Faith PD), MC-I had significantly higher values than MC-II (*p* < 0.0001) and MC-III (*p* = 0.0109), while MC-V showed significantly higher phylogenetic diversity than both MC-II (*p* < 0.0001) and MC-III (*p* = 0.0303). For observed features, MC-I showed significantly higher richness than MC-II (*p* < 0.0001) and MC-III (*p* = 0.0121). MC-IV and MC-V also had significantly higher values than MC-II (*p* = 0.0181 and *p* < 0.0001, respectively), and MC-V was significantly higher than MC-III (*p* = 0.0479). In Shannon entropy, MC-I exhibited significantly higher values than MC-II (*p* < 0.0001), MC-III (*p* = 0.0032), and MC-IV (*p* = 0.0015). MC-III, MC-IV, and MC-V also showed significantly higher entropy than MC-II (*p* = 0.0026, *p* = 0.0413, and *p* < 0.0001, respectively). For the Simpson index, MC-I showed significantly higher diversity than MC-II (*p* < 0.0001), MC-III (*p* = 0.0144), and MC-IV (*p* < 0.0001). MC-III and MC-V were also significantly higher than MC-II (*p* < 0.0001 and *p* = 0.0001, respectively). For Simpson evenness, MC-I exhibited significantly higher evenness than MC-II (*p* < 0.0001), MC-IV (*p* < 0.0001), and MC-V (*p* = 0.0029). MC-III also had significantly higher values than MC-II (*p* = 0.0003) and MC-IV (*p* = 0.0033). No other comparisons were statistically significant across the indices.

**Table 2:** The α-diversity in five substrate groups (MC-I to MC-V).

**Alpha diversity metric**	**MC-I**	**MC-II**	**MC-III**	**MC-IV**	**MC-V**	***p*-value**
Chao1	301.92 ± 111.59^a^	149.33 ± 36.32^b^	203.65 ± 85.67^ab^	236.05 ± 73.46^a^	325.04 ± 113.76^a^	< 0.0001
Pielou_evenness	0.88 ± 0.03^a^	0.69 ± 0.04^c^	0.79 ± 0.03^b^	0.75 ± 0.04^bc^	0.81 ± 0.03^ab^	< 0.0001
Faith_pd	14.35 ± 2.82^a^	9.43 ± 1.4^b^	10.74 ± 2.32^b^	11.6 ± 2^ab^	14.64 ± 2.76^a^	< 0.0001
Observed_features	230.13 ± 68.14^a^	118.5 ± 27.25^c^	153.24 ± 47.97^bc^	172.13 ± 37.64^ab^	226.67 ± 53.18^a^	< 0.0001
Shannon_entropy	6.86 ± 0.63^a^	4.76 ± 0.46^c^	5.67 ± 0.6^b^	5.54 ± 0.46^b^	6.33 ± 0.47^ab^	< 0.0001
Simpson	0.98 ± 0.01^a^	0.9 ± 0.02^c^	0.95 ± 0.02^b^	0.93 ± 0.02^bc^	0.96 ± 0.01^ab^	< 0.0001
Simpson_evness	0.24 ± 0.1^a^	0.07 ± 0.02^c^	0.12 ± 0.05^ab^	0.07 ± 0.02^c^	0.09 ± 0.03^bc^	< 0.0001

Average value with standard deviation (SD). Values with differing letters were significantly different, as determined by Dunn's Multiple Comparison Test (*p*-value < 0.005).

Fermentation profiles for each MC group are shown in Table 3, and those for each substrate are presented in Supplementary Table S4. MC-I exhibited a significantly higher pH than MC-II and MC-III (*p* < 0.0001), while MC-II showed significantly lower pH than MC-IV and MC-V (*p* = 0.0052 and 0.0064, respectively). MC-I produced significantly less total gas than MC-II, MC-III, and MC-IV (*p* < 0.0001, *p* < 0.0001, and *p* = 0.0166, respectively), while MC-V produced significantly less gas than MC-II and MC-III (*p* = 0.0013 and *p* = 0.0002, respectively). Total SCFA concentrations were significantly lower in MC-I compared to MC-II, MC-III, and MC-IV (*p* < 0.0001, *p* < 0.0001, and *p* = 0.0052, respectively), and in MC-V compared to MC-II and MC-III (*p* = 0.0021 and *p* = 0.0013, respectively). Acetate, propionate, and butyrate concentrations were consistently lower in MC-I than in MC-II, MC-III, and MC-IV (all *p* < 0.01), with MC-V also showing significantly lower levels than MC-II or MC-III in most comparisons (acetate: *p* = 0.0014 and *p* = 0.0021; propionate: *p* = 0.0042 and *p* = 0.0002; butyrate: *p* = 0.0088 and *p* = 0.0106). MC-II had a higher acetate proportion than MC-III (*p* = 0.0352), and MC-III had a higher propionate proportion than MC-I (*p* = 0.0373). Butyrate proportion was significantly higher in MC-V (*p* = 0.0067) and MC-III (*p* = 0.0345) than in MC-IV. Notably, lactate accumulation was pronounced in MC-II (4.87 ± 2.33 mmol/L), which was significantly higher than in all other groups (MC-I: *p* < 0.0001; MC-III: *p* = 0.0007; MC-IV and MC-V: *p* < 0.0001). Although lactate was also detected in MC-III at low levels, its concentration did not differ significantly from those in MC-I, MC-IV, or MC-V. Similarly, formate was only detected in MC-II (0.99 ± 1.16 mmol/L), which showed significantly higher concentrations than all other groups (MC-I: *p* < 0.0001; MC-III: *p* < 0.0001; MC-IV: *p* < 0.0001; MC-V: *p* = 0.0015). All other pairwise comparisons were not statistically significant.

**Table 3:** Organic acid production, pH, and gas production are dependent on substrate and ruminal microbial community composition.

**Parameter**	**MC-I**	**MC-II**	**MC-III**	**MC-IV**	**MC-V**
pH	6.81 ± 0.06^a^	6.49 ± 0.15^c^	6.59 ± 0.09^bc^	6.68 ± 0.08^ab^	6.71 ± 0.06^ab^
Total gas (ml)	0.89 ± 0.45^d^	4.83 ± 0.92^ab^	5.90 ± 2.59^a^	2.83 ± 0.73^bc^	1.64 ± 0.42^cd^
Total SCFAs (mmol/L)	20.73 ± 1.76^c^	39.74 ± 6.87^a^	42.82 ± 13.19^a^	30.51 ± 5.97^ab^	24.14 ± 1.67^bc^
Acetate (mmol/L)	14.45 ± 1.20^c^	26.57 ± 5.34^a^	26.19 ± 6.79^a^	20.54 ± 4.24^ab^	16.31 ± 1.01^bc^
Propionate (mmol/L)	4.18 ± 0.46^d^	9.90 ± 1.73^ab^	12.91 ± 5.39^a^	7.43 ± 1.50^bc^	5.37 ± 0.76^cd^
Butyrate (mmol/L)	2.10 ± 0.21^b^	3.27 ± 0.28^a^	3.72 ± 1.16^a^	2.54 ± 0.29^b^	2.46 ± 0.26^b^
Acetate (%)	69.73 ± 0.84^a^	66.58 ± 3.42^b^	62.01 ± 3.46^c^	67.21 ± 1.13^b^	67.60 ± 1.23^ab^
Propionate (%)	20.14 ± 1.22^c^	25.07 ± 3.02^ab^	29.28 ± 3.56^a^	24.33 ± 0.92^b^	22.21 ± 2.15^bc^
Butyrate (%)	10.13 ± 0.49^a^	8.35 ± 0.80^b^	8.71 ± 0.46^b^	8.46 ± 0.88^b^	10.19 ± 1.00^a^
A/P	3.48 ± 0.24^a^	2.71 ± 0.45^b^	2.16 ± 0.38^c^	2.77 ± 0.13^b^	3.07 ± 0.33^ab^
Lactate (mmol/L)	0 ± 0^b^	4.87 ± 2.40^a^	1.35 ± 2.40^b^	0 ± 0^b^	0 ± 0^b^
Formate (mmol/L)	0 ± 0^b^	0.99 ± 1.20^a^	0 ± 0^b^	0 ± 0^b^	0 ± 0^b^

Average value with standard deviation (SD). Values with differing letters were significantly different, as determined by Dunn's Multiple Comparison Test (*p*-value < 0.005).

## 4 Discussion

To explore novel woody feed resources, we conducted an *in vitro* fermentation experiment using a broad range of substrates—including steamed and untreated woods, lignocellulosic feed by-products, oligosaccharides, and conventional feeds—and analyzed their respective impacts on rumen fermentation profiles and microbial community structures. Clustering analysis based on these data revealed substrate-specific fermentation and microbial patterns (Figure 1). Envfit analysis indicated that overall fermentability, reflected by total SCFA concentration and gas production, was significantly associated with variations in microbial community structure. However, these fermentation parameters were more strongly associated with a secondary axis of variation rather than the primary axis, which accounted for the largest proportion of community dissimilarity, indicating that fermentability alone does not fully explain the main patterns of microbial separation across substrates.

We found that the bacterial community was clustered into five substrate groups (MC-I–MC-V). Within MC-I, the classification included blanks or non-steamed woods which had significantly lower total SCFAs and gas production compared to other groups except MC-V (Table 3). These results align with earlier findings indicating that untreated ground wood is poorly fermented in the rumen. In a study by Hamano et al. (30), micronized Japanese cedar ground wood resulted in only limited VFA production *in vitro*. Consistently, *in vivo* feeding of this material did not enhance ruminal fermentation. These outcomes highlight the recalcitrant nature of unprocessed lignocellulosic substrates and reinforce our interpretation of MC-I as a non-fermenting microbial cluster. This group shows low α-diversity (Table 2). A possible explanation for this reduced diversity is the limited availability of substrates utilizable by bacteria. Indeed, other studies have reported that microbial communities tend to exhibit lower diversity under conditions where substrate availability is highly restricted or compositionally narrow (31). Although no specific biomarkers were identified by LEfSe analysis in MC-I, *Prevotella* was the most dominant genus in this group, accounting for approximately 50% of the microbial composition (Supplementary Table S3). The lack of identified biomarkers in MC-I and MC-V may reflect biological heterogeneity and statistical constraints, as high within-cluster variability relative to sample size could limit the detection of consistent biomarkers. *Prevotella* is one of the predominant genera in ruminants and is known for its resilience to environmental fluctuations (32, 33). This ecological stability may explain its high relative abundance in the MC-I group, where limited availability of fermentable substrates likely led to minimal shifts in the bacterial community composition.

MC-II was primarily composed of xylo-oligosaccharides and also contained rice straw. Xylo-oligosaccharides are produced, particularly xylan, during the hydrolysis of hemicellulosic materials (34). *Selenomonas* was identified by LEfSe analysis as being significantly more abundant in MC-II, and species such as *S. ruminantium* has been shown to degrade xylo-oligosaccharides derived from xylan via a broad-specificity xylosidase/arabinosidase enzymes, thus contributing to hemicellulose breakdown in the rumen (35). Although the xylo-oligosaccharides used in this study were not derived from rice straw, rice straw itself is known to contain high levels of xylo-oligosaccharides. Furthermore, previous studies have reported that xylo-oligosaccharides selectively promote the growth of certain microbial populations in the human gut (36). Notably, previous studies have reported that xylo-oligosaccharides can selectively promote the growth of specific microbial taxa in the gut (36), and in our study, the dominance of xylo-oligosaccharide-degrading bacteria such as *Selenomonas* was also observed in this cluster. Such selective nutrient enrichment may have favored the proliferation of certain microbes while limiting the growth of others, thereby constraining the overall microbial diversity. Indeed, microbial communities are known to exhibit lower diversity under conditions where available substrates are chemically simple or structurally restricted (37, 38).

MC-III was predominantly classified with common feed ingredients such as concentrate and timothy hay. These substrates are known to contain not only structural carbohydrates but also readily fermentable carbohydrates such as starch. In line with this, *Streptococcus*—a genus known for its capacity to degrade soluble carbohydrates including starch (39)—was found to be significantly more abundant in MC-III. Given that the substrates used in the other groups were primarily composed of hemicellulose-derived components such as xylo-oligosaccharides and steamed wood, the presence of *Streptococcus* in MC-III may reflect the relatively higher availability of starch and other soluble sugars in this group.

The MC-IV group was characterized by white-birch wood oligosaccharide, steamed white-birch, fermented bagasse, bamboo-culm oligosaccharide, and spent *L. edodes* substrate. The spent mushroom substrate was likely included in this group as birch was incorporated in the initial *L. edodes* substrate. Some oligosaccharides (white-birch wood oligosaccharide, bamboo-culm oligosaccharide) were classified into MC-IV rather than MC-II. White-birch wood contains abundant xylan, with a reported structure consisting of xylose, acetyl groups, and 4-O-methylglucuronic acid in a molar ratio of approximately 15:6:1, showing a high prevalence of acetyl substitutions (40). These structural features of birch-wood derived hemicellulose may create a selective ecological niche, promoting the proliferation of *Succinivibrio* spp., which were significantly abundant in MC-IV. Although *Succinivibrio* is not a major fibrolytic bacterium, it can utilize hemicellulosic substrates like xylan (41, 42). Despite its limited growth on simple xylo-oligosaccharides (43), its increased abundance here suggests that structural features of birch-derived oligosaccharides—such as acetylation and side chains—may have supported its proliferation and shaped the microbial community. Therefore, the structural differences in this hemicellulose especially its acetylation and side-chain composition may have influenced the increased abundance of *Succinivibrio* and contributed to the shaping of the bacterial community.

In MC-V, steamed feeds excluding white-birch wood were classified, but no specific biomarker bacteria were detected. Steamed wood formed a distinct cluster from wood meal (MC-I) and common feed (MC-III) (Figure 2). Although the fermentation characteristics of MC-V were similar to those of untreated wood (MC-I), their distinct clustering patterns suggest that steam treatment altered the types of carbohydrates available to microbes, thereby modifying microbial community structure. This is consistent with findings by Shimizu et al. (44), who reported that steam treatment changes the composition and enzymatic susceptibility of hemicellulosic polysaccharides. Furthermore, that MC-V formed a separate cluster from MC-III, which included conventional feed materials, indicates that steam treatment exerts a unique influence on the rumen microbiota, distinct from that of common feed. However, this influence differed from that observed in MC-IV, which contained steamed white-birch wood.

The present results suggest that microbial community patterns across the tested substrates were mainly associated with differences in carbohydrate structure, particularly hemicellulose composition and modification. In addition to these structural carbohydrate effects, previous studies have reported that certain plant-derived compounds, such as triterpenoids including betulin, can influence specific ruminal bacteria (45). Although such compounds were not assessed in the present study and their behavior during steam treatment remains unclear, considering these effects may be useful in future studies. Moreover, it should be noted that *in vitro* batch culture systems have inherent limitations, as they do not account for nutrient absorption, host–microbe interactions, or long-term microbial adaptation. Accordingly, the responses observed here should be interpreted strictly as substrate-specific effects under controlled *in vitro* conditions, and further *in vivo* studies will be necessary to evaluate their relevance under practical feeding conditions.

## 5 Conclusion

This study demonstrated that both steamed wood and xylo-oligosaccharides create distinct rumen fermentation profiles and bacterial community structures under *in vitro* conditions, depending on the substrate.

While untreated wood exhibited limited fermentability and minimal impact on microbial composition, steam-treated wood and xylo-oligosaccharides each led to the formation of microbial clusters dominated by specific genera such as *Succinivibrio* and *Selenomonas*, respectively, and had unique fermentation patterns. Structural features of steam-derived hemicellulose and oligosaccharides are thought to contribute to these microbial shifts. These findings suggest that steam-treated wood and xylo-oligosaccharides have potential as sustainable feed ingredients and may influence rumen fermentation and microbial community structure under *in vitro* conditions. Further *in vivo* studies are needed to evaluate their efficacy and long-term impacts on animal health and production performance.

## Data Availability

The datasets presented in this study can be found in online repositories. The names of the repository/repositories and accession number(s) can be found below: https://www.ddbj.nig.ac.jp/, DRR699410–DRR699487.
